# Pharmacokinetic and Efficacy Study of Acyclovir Against Cyprinid Herpesvirus 3 in *Cyprinus carpio*

**DOI:** 10.3389/fvets.2020.587952

**Published:** 2020-10-22

**Authors:** Eva Marie Quijano Cardé, Zeinab Yazdi, Susan Yun, Ruixue Hu, Heather Knych, Denise M. Imai, Esteban Soto

**Affiliations:** ^1^Department of Medicine and Epidemiology, School of Veterinary Medicine, University of California, Davis, Davis, CA, United States; ^2^K. L. Maddy Equine Analytical Pharmacology Laboratory, School of Veterinary Medicine, University of California, Davis, Davis, CA, United States; ^3^Comparative Pathology Laboratory, University of California, Davis, Davis, CA, United States

**Keywords:** antiviral therapy, cidofovir, fish medicine, KHV, koi Acyclovir

## Abstract

Cyprinid Herpesvirus 3 (CyHV-3), more commonly known as Koi Herpesvirus (KHV), is a re-emergent virus causing acute systemic infection with high mortality rates in koi fish (*Cyprinus carpio*). Survivors from outbreaks can become latent carriers, with viral reactivation under stressful conditions and permissible temperatures. No vaccines or treatments are currently available in the United States. Acyclovir has been shown effective *in vitro* against KHV. This study aimed to evaluate the cytotoxicity of acyclovir and cidofovir to koi fin (KF1) cells, the efficacy of a single antiviral intracoelomic dose in a koi fingerling cohabitation challenge, and the pharmacokinetics of the effective antiviral. Initially, a lactate dehydrogenase release-based assay revealed no significant acyclovir or cidofovir cytotoxicity to KF1 cells for 24 h at up to 1,500 μM. In laboratory-controlled challenges, KHV associated mortalities occurred 2 weeks post-infection. At this point, fish were treated with an antiviral (10 mg/kg acyclovir or 5 mg/kg cidofovir) or sterile phosphate-buffered solution. Morbidity and mortality were monitored for 30 days. A significant cumulative mortality reduction (*p* ≤ 0.05), and a 3-day mortality delay were detected in the acyclovir-treated group. Similar viral loads were detected in gills recovered from mortalities throughout the challenge and surviving fish at the end of the challenge regardless of treatment. For pharmacokinetic analysis, blood was collected at various timepoints after acyclovir administration. Liquid chromatography tandem mass spectrometry plasma analysis indicated a 141 μM peak plasma concentration at 0.75 h, a 14 h half-life, and a 0.05/h elimination rate constant. Histopathology of target tissues detected no evidence of acyclovir toxicity. Results suggest that a single 10 mg/kg dose of acyclovir administered intracoelomically to koi fingerlings is safe and reduces cumulative mortality during a KHV mortality event. However, multiple doses are probably required for effective treatment of pet fish.

## Introduction

Freshwater fishes are the most numerous pet in the United States (US), with ~139 million fishes reported throughout 12 million households (1). Koi carp fish, the ornamental variety of the common carp (*Cyprinius carpio*), are among the most popular freshwater ornamental fishes worldwide (2). While koi are among some of the hardiest fishes in the ornamental trade, seasonal mass mortalities were documented in the US, Japan, and other countries starting in the mid 1990's. In 2000, the causative agent was identified to be a herpesvirus that was later named Cyprinid Herpesvirus 3, also known as Koi Herpesvirus (KHV) (3).

This virus can infect goldfish (*Carassius auratus*), crucian carp (*Carassius carassius*), common carp, and koi carp, but only causes disease in common carp and koi carp, leading in some cases to death in <21 days in over 80% of the infections (4). Similar to other Cypriniviruses, KHV diseases is a temperature-dependent disease that causes clinical signs over the range of 15–28°C (5). Exposing fish to this virus or changing the temperature post-infection to above or below the permissive range has been shown to reduce or even eliminate mortalities (6). Because of this temperature dependency, temperature modifications have been used therapeutically. Unfortunately, typical of herpesviruses, KHV undergoes latency and is able to become active when temperatures return to permissive (7). While exposed carriers can be more resistant to subsequent outbreaks and reduced mortality is seen in some cases (8), naïve fish infected from these carriers still have the same morbidity and mortality rates. Currently, there are no treatments or vaccines available in the US against KHV infections. An attenuated live vaccine and an inactivated vaccine have been developed in other countries such as Israel, but risk of reactivation and low protection have hampered their use in the US (4).

Previous studies have looked into the effects of antivirals against KHV. While the *Clinacanthus nutans* extract has been reported to decrease fish mortality when administered up to 24 h post-infection (9), this compound is not regulated and thus not available in a commercial formulation. The deoxyguanosine analog acyclovir, which is capable of halting viral replication via selective inhibition of the viral DNA polymerase, has been reported to decrease *in vitro* cytopathic effects, viral load, and viral gene expression when the antiviral was added 2 h post-infection (10). Acyclovir is an approved antiviral by the US Food and Drug Administration (FDA) for the treatment of Herpes Simplex Virus 1 and 2 in humans and hence a good option for extralabel use in pet fish under veterinary care. Cidofovir, an FDA-approved acyclic phosphonate nucleotide analog, was selected as a candidate antiviral as well for its reported success against both human herpesviruses and feline herpesvirus 1 (11, 12). In contrast to acyclovir, cidofovir does not require the initial phosphorylation step, which is achieved by a viral-encoded thymidine kinase, and it has been reported to have secondary active metabolites with longer half-lives in humans (13).

Therefore, this study aimed to evaluate the cytotoxicity of acyclovir and cidofovir to koi fin (KF1) cells and to determine the efficacy of these antivirals in decreasing cumulative mortality during a KHV challenge in koi fingerlings. Additionally, this study aimed to determine the plasma pharmacokinetics of a single acyclovir intracoelomic injection in koi fingerlings.

## Materials and Methods

### Cell Cultures

KF1 cells were grown from stocks and maintained in Minimum Essential Media (MEM; Corning Inc., Corning, NY) supplemented with 7.5% Fetal Bovine Serum (FBS), L-glutamine, and Penicillin/Streptomycin (Genesee Scientific, San Diego, CA) at 20°C.

### Antivirals

Acyclovir was obtained in powder form from Fisher Scientific (Waltham, MA), while cidofovir was obtained in powder form from Millipore Sigma (Burlington, MA). Stock solutions were made in sterile Phosphate-Buffered Solution (PBS) and used within 1 h.

### Antiviral-Induced Cytotoxicity Assay

Promega's CytoTox 96 Non-radioactive Cytotoxicity Assay (Promega Corporation, Madison, WI) with some modifications was used to assess the cytotoxicity of acyclovir and cidofovir in KF1 cells. Briefly, KF1 cells were plated on duplicate 96-well plates at 90–100% confluency in MEM 2% FBS + HEPES and allowed to attach for 24 h. Each antiviral was tested in separate plates for a total of two technical replicates per antiviral. Triplicate wells of various concentrations (5–1,500 μM) were made using the stock solutions and the cells were incubated for 24 h. Lactate dehydrogenase release was measured via absorbance. Negative and positive controls were included, where positive controls were optimized by an 8-h incubation time with the lysis solution. Cytotoxicity was calculated as a percentage of the appropriate positive control.

### Fish

A group of 800 koi fingerlings weighing 3 grams in average were obtained from a commercial koi breeder with no history of KHV or Cyprinid Herpesvirus 1 infections and allowed to acclimatize to the 259-gallon circular holding tank for 2 months in a flow-through system between 16 and 17°C. A random sample of six koi fingerlings was selected for general health assessment and gill clip KHV testing using conventional polymerase chain reaction (PCR) as described in Gilad et al. (14). In addition, euthanasia with 1,000 ppm buffered tricaine methanesulfonate (MS222, 1:1 sodium bicarbonate; Syndel USA, Ferndale, WA) was performed on this fish subset to collect heart and kidney samples for bacterial culture. During this time, the remaining koi fingerlings were fed 4% of their body weight daily via an automatic feeder of 1 mm-salmon sink pellet feed (Skretting: a Nutreco company, Stavanger, Norway). Temperature was monitored daily and dissolved oxygen measured weekly. All protocols and procedures using these koi fingerlings were ethically reviewed and approved by the University of California Institutional Animal Care and Use Committee.

### Virus Culture

KF1 cells were plated on 6-well plates at approximately 85% confluency in MEM 2% FBS + HEPES. A KHV isolate, previously recovered from an outbreak in California, US (strain ID CDFG08-70 UCD_CA29 MK804584, Clear Lake carp), was propagated from stock in KF1 cells by means of subculture.

### Viral Plaque Assay

A standard plaque assay with some modifications was used to determine plaque forming units (pfu) per mL. Briefly, KF1 cells were plated on 24-well plates at 90–100% confluency in MEM 2% FBS + HEPES and allowed to attach for 24 h. Duplicate wells of various dilutions were made for the KHV isolate. MEM 2% FBS+ HEPES media with 0.75% methylcellulose was used as a semi-solid medium for plaque formation and the infected cells were incubated for 10 days at 20°C. A 0.6% crystal violet solution (10 μL per well) was used for staining and plaques were counted manually via microscopy.

### Cohabitation Challenge

A group of 420 koi fingerlings was used in total for the challenge experiment, averaging 8 g in weight. A subgroup of 30 koi fingerlings was exposed to a KHV 0.6 pfu/mL bath for 6 h (15). Fish were monitored during the following 9 days to allow for infection and were then anesthetized using 100 ppm buffered MS222. A fin clip of the dorsal aspect of the caudal fin was performed (*n* = 29) to estimate prevalence of infection in the group via quantitative PCR (qPCR) (6). Fish were then divided among the experimental infected groups for a cohabitation model (16), which represents more accurately a natural outbreak. This led to three infected groups of 100 koi fingerlings each (1:10 ratio, infected:naïve fish). Clipping a small portion of the dorsal caudal fin also allowed differentiation of infected animals and naïve fish. Another subgroup of 30 koi fingerlings was exposed to the same volume of sterile MEM 2% FBS + HEPES culture media for 6 h to be used in the negative control group. Only 10 of these koi fingerlings were added to form the negative control group of 100 koi fingerlings. Fish were then maintained at 16–17°C in 35-gallon tanks, fed 1% of their body weight of 1 mm-salmon sink pellet feed (Skretting: a Nutreco company, Stavanger, Norway) once daily, and monitored closely. All mortalities (*n* = 7) were confirmed KHV positive via qPCR as previously described.

After the first mortality from cohabitation exposure was confirmed KHV positive, fish were anesthetized using 100 ppm buffered MS222. Weight was obtained for each fish and the appropriate drug was administered via an intracoelomic injection just cranial to the pelvic fins (10 mg/kg acyclovir, 5 mg/kg cidofovir, and 10 mL/kg PBS) using a 1 mL syringe with a 22g needle. The cidofovir stock solution concentration was diluted to account for the lower dose to maintain the volume injected uniform among groups. Drug doses were selected based on intravenous protocol recommendations for acyclovir and cidofovir in humans (13, 17). Random fin clip sampling was performed to estimate prevalence of infection in the group as previously described (*n* = 20). The koi fingerlings were recovered in separate 5-gallon tanks forming 6 replicate tanks with 15 fish each for each group.

Mortality was then recorded for 30 days and gills collected from mortalities for KHV status confirmation and viral load assessment via qPCR (*n* = 17, 16, and 13 for the positive control, acyclovir, and cidofovir, respectively). At the end of the 30 days, survivors were euthanized with 1,000 ppm buffered MS222 and gills of 12 koi fingerlings per group were sampled assess viral load and final prevalence of KHV after the outbreak (6).

### DNA Sample Generation

The DNeasy Blood & Tissues kit (QIAGEN, Hilden, Germany) was used to extract DNA from samples kept at −80°C, following the manufacturer's recommendation. DNA was eluted in 100 μL of elution buffer.

### Viral Load Quantification

Quantitative PCR using the QuantStudio3 qPCR System (Thermo Fisher Scientific, Waltham, MA) was performed to evaluate viral load throughout the challenge experiment following published protocols (6).

### Pharmacokinetic Study

A group of 102 koi fingerlings averaging 9.8 g in weight were used for the pharmacokinetic study. Anesthesia was induced with 100 ppm buffered MS222 and a 10 mg/kg acyclovir dose was administered intracoelomically to each fish just cranial to the pelvic fins. Groups were recovered and separated in 5-gallon tanks, forming 16 timepoints of six koi fingerlings each (0.25, 0.5, 0.75, 1, 2, 4, 6, 8, 12, 24, 36, 48, 72, 96, 120, and 144 h post-injection). In addition, a negative control group was added for generation of the acyclovir standard curve as well as assessment of normal organ histopathology.

At the appropriate time post-drug administration, fish were euthanized with 1,000 ppm buffered MS222. Blood was collected from the caudal tail vein into lithium heparin-lined capillary tubes after severing the caudal peduncle with a #10 scalpel blade. Tubes were centrifuged (IEC MB centrifuge, 5 min at 16,000 RCF) and plasma stored at −80°C until processing. A full necropsy was then performed to collect samples of the spleen, gills, brain, liver, heart, and kidney for histopathology analysis.

For determination of plasma drug concentration, acyclovir working solutions were prepared by dilution of the stock solution (Cerilliant, Round Rock, TX) with methanol. Plasma calibrators were prepared by dilution of the working standard solutions with drug free fish plasma. Calibration curves and negative control samples were prepared fresh for each quantitative assay. In addition, quality control samples (fish plasma fortified with analyte at two concentrations within the standard curve) were included as an additional check of accuracy.

Prior to analysis, 20 μL of plasma were diluted with 150 μL of ACN:1M acetic acid (9:1, v:v) containing 10 ng/mL of the internal standard penciclovir (AK Scientific, Union City, CA) to precipitate proteins. The samples were vortexed for 1 min to mix, refrigerated for 20 min, vortexed for an additional 0.5 min, and centrifuged (3,102 g) for 10 min at 4°C. After protein precipitation and prior to analysis, the supernatant was transferred to an autosampler vial with an insert and then 30 μL were injected into the liquid chromatography tandem mass spectrometry (LC-MS/MS) system.

The acyclovir concentration was measured in plasma by LC-MS/MS using positive heated electrospray ionization. Quantitative analysis was performed on a TSQ Altis triple quadrupole mass spectrometer coupled with a Vanquish liquid chromatography system (Thermo Scientific, San Jose, CA). The spray voltage was 3,500 V, the vaporizer temperature was 350°C, and the sheath and auxiliary gas were 50 and 10, respectively (arbitrary units). Product masses and collision energies of each analyte were optimized by infusing the standards into the TSQ Altis. Chromatography employed an Atlantis HILIC 15 cm x 2.1 mm 5 μm column (Waters Corp, Milford, MA) and a linear gradient of acetonitrile (ACN) in water with a constant 0.2% formic acid at a flow rate of 0.5 ml/min. The initial ACN concentration was held at 95% for 0.5 min, ramped to 5% over 4.5 min, and held at that concentration for 0.1 min before re-equilibrating for 2.4 min at initial conditions.

Detection and quantification were conducted using selective reaction monitoring of initial precursor ion for acyclovir [mass to charge ratio (*m/z*) 226] and the internal standard penciclovir (*m/z* 254). The response for the product ion for acyclovir (*m/z* 152), and the internal standard (*m/z* 135, 152), were plotted and peaks at the proper retention time integrated using Quanbrowser software (Thermo Scientific). Quanbrowser software was used to generate calibration curves and quantitate analytes in all samples by linear regression analysis. A weighting factor of 1/X was used for all calibration curves.

The concentration-response relationship (relationship between calibrators and the LC-MS/MS instrument response) for acyclovir was linear (*R* = 0.99). The precision and accuracy of the assay was determined by assaying quality control samples in replicates (*n* = 6). Accuracy was reported as percent nominal concentration and precision was reported as percent relative standard deviation. For acyclovir, accuracy was 99% for 0.013 μM and 87% for 1.11 μM. Precision was 13% for 0.013 μM and 3% for 1.11 μM. The technique was optimized to provide a limit of quantitation of 0.004 μM and a limit of detection of ~0.002 μM for acyclovir.

Pharmacokinetic parameters were calculated by non-compartmental analysis of sparse data, in which the plasma drug concentrations from all fish were analyzed simultaneously. Standard errors of the mean for AUC_last_ and C_max_ values were calculated as described in Nedelman and Jia (18), using a modification in Holder (19).

### Statistical Data Analysis

A nested one-way ANOVA and a Dunnett's *post-hoc* analysis were implemented for determination of statistical significance of the cytotoxicity assay data. A one-way ANOVA and a Tukey's *post-hoc* analysis with a single pooled variance were implemented for statistical significance determination of the viral load data, except for the comparison of mortalities to survivors' viral load with a two-tailed nested *t*-test. A Kruskal-Wallis test and a Dunn's *post-hoc* analysis were implemented for statistical significance determination of the time to first mortality data. The aforementioned analyses were performed using GraphPad Prism 8 [Version 8.4.2 (464), GraphPad Software LLC].

The SAS® (Version 9.4, SAS Institute, Cary NC) mixed procedure was used to analyze the cumulative mortality data as a repeated measures analysis of variance in a 4 × 30 factorial arrangement of treatments. Factors in the model included treatment (negative control, positive control, acyclovir, and cidofovir) and day (1–30). The random effect in the model was tank within treatment. The response variable was the percent cumulative mortality. When overall significance was detected for main effects or interaction effects, *post-hoc* comparisons were conducted with pairwise *t*-tests of least-squares means.

All comparisons were considered significant at *p* ≤ 0.05.

## Results

### Cytotoxicity Assay

**Figure 1:**
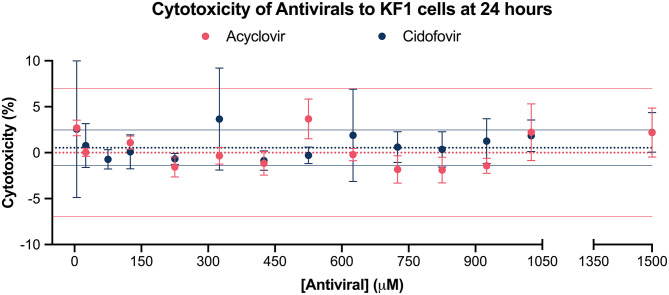
Cytotoxicity of acyclovir and cidofovir to KF1 cells at 24 h. Antiviral cytotoxicity on the koi fin cell line (KF1) was evaluated by exposing the cells to concentrations from 5 μM to 1,500 μM for 24 h and measuring the release of lactate dehydrogenase in antiviral-treated cells, negative controls, and positive controls. The solid dots represent the mean cytotoxicity percentage at each exposed concentration, with standard error of the mean plotted. The dotted line represents the mean cytotoxicity percentage of the negative control, while the solid lines represent its 95% confidence interval. The different experimental groups are color coded. Results suggest that both acyclovir and cidofovir at the tested concentrations are non-cytotoxic to KF1 cells when compared to the negative control.

There was no statistically significant difference in cytotoxicity to KF1 cells between the acyclovir or cidofovir treated groups and the negative controls, as determined by the Promega's CytoTox 96 Non-radioactive Cytotoxicity Assay (Figure 1).

### Challenge Assay

**Figure 2:**
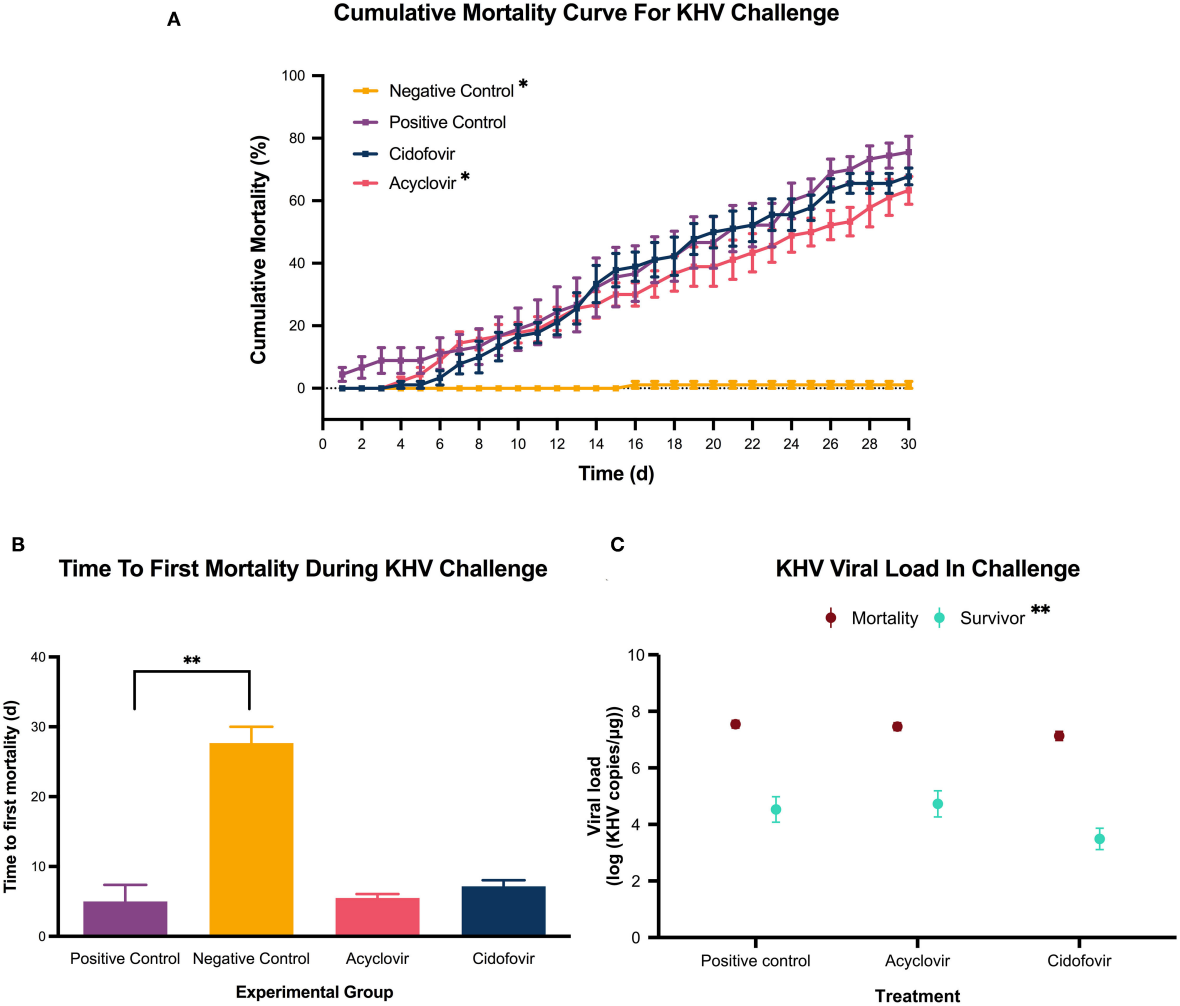
KHV challenge. A cohabitation challenge model was performed by exposing subgroups of koi fingerlings to a 0.6 KHV pfu/mL bath or a sterile cell culture media bath for 6 h. Once mortalities were detected and KHV confirmed via qPCR, groups were administered a single 10 mg/kg acyclovir intracoelomic dose, a single 5 mg/kg cidofovir intracoelomic dose, or a 10 mL/kg sterile PBS solution intracoelomic injection. Mortality was monitored for 30 days after drug administration. **(A)** The solid squares represent the mean cumulative mortality at each day, with standard error of the mean plotted. The different experimental groups are color coded. The black stars indicate statistical significance when compared to the positive control with a *p* ≤ 0.05. A statistically significant reduction in cumulative mortality (days 25–30 for the acyclovir-treated group, and days 7–30 for the negative control group) was detected when compared with the positive control group. A delay in mortality. Similarly, the negative control cumulative mortality was statistically significantly lower when compared with the positive control group (days 7–30). A delay in mortality was observed in both the acyclovir and cidofovir groups with the first mortality occurring 4 days post-treatment, compared to 1 day in the positive control group. **(B)** Each bar represents the mean time to first death in days for the color-coded group, with standard error of the mean plotted. The black stars indicate statistical significance when compared to the positive control with a *p* = 0.0013. **(C)** The solid dots represent the mean viral load, with the standard error of the mean plotted. The different sampled groups are color coded. The black stars indicate statistical significance with a *p* = 0.0013. There was no statistically significant difference in viral load among the treatment groups, but results reveal a statistically significant reduction in mean viral load in survivors of the challenge compared to the challenge mortalities.

The prevalence of KHV infection was 79.3% 9 days post-immersion challenge, 55% after cohabitation (before treatment administration), and 100% at the end of the 30-day challenge assay. All tested negative controls were KHV negative. Analysis of cumulative mortality revealed a statistically significant (*p* ≤ 0.05) 15% mean reduction in cumulative mortality from days 25 to 30 in the acyclovir-treated group when compared to the positive control. There was no statistically significant reduction in cumulative mortality in the cidofovir group when compared to the positive control. In addition, there was a 3-day delay in first mortality in the acyclovir and cidofovir groups when compared to the positive control group. The positive control had a 76% cumulative mortality by day 30 (Figure 2A). Only 1 fish from the negative control group died during the challenge assay and was confirmed KHV negative via qPCR; the cause of death of this individual was not identified.

Given the delay in first mortality in antiviral-treated groups, time to first death per group was assessed. There was no statistical significance between the time to first death in antiviral-treated groups when compared to the positive control group (Figure 2B).

The molecular data from the challenge assay revealed a similar viral load in the infected experimental groups for the tested mortalities as well as the tested survivals (Figure 2C). However, when comparing the nested viral loads of the challenge infected mortalities with the infected survivals, there was a statistically significant (*p* = 0.0013) decrease in viral load from a mean of 7.4 log_10_(KHV copies/μg) in mortalities to 4.2 log_10_(KHV copies/μg) in survivals (Figure 2C).

### Pharmacokinetic Analysis

**Figure 3:**
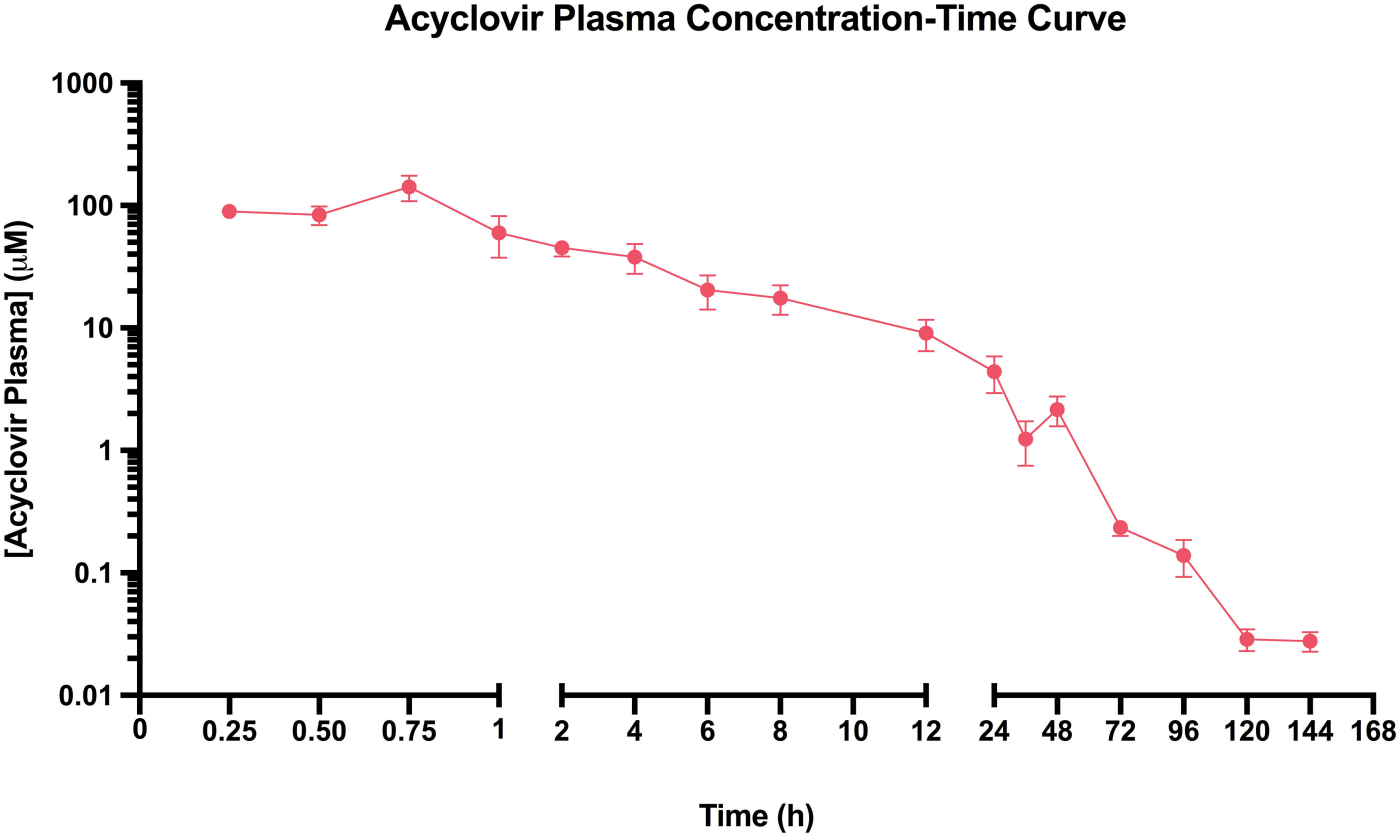
Acyclovir Plasma Concentration-Time Curve. Koi fingerlings received a single 10 mg/kg acyclovir intracoelomic dose. Blood from the caudal vein was collected after euthanasia at different timepoints from 15 min to 6 days after drug administration. The solid pink dots represent the mean acyclovir plasma concentration at each time-point, with standard error of the mean plotted. Results indicate that an acyclovir peak plasma concentration of 141 μM is obtained in 0.75 h, with a half-life of 14 h.

The plasma concentration-time curve indicates an acyclovir peak plasma concentration of 141 μM obtained at 0.75 h (Figure 3). The elimination rate constant calculated was 0.05/h and the half-life was 14 h (Table 1).

**Table 1:** Plasma pharmacokinetic parameters of acyclovir in koi fingerlings receiving a single 10 mg/kg intracoelomic dose (*n* = 6 fish per time point).

**Parameter**	**Units**	**Estimate**
Elimination rate constant (λ_z_)	1/h	0.05
Terminal half-life (HL- λ_z_)	h	14
Maximum concentration (C_max_)	μM	141.49 ± 33^†^
Time to maximum concentration (T_max_)	h	0.75
Area under the curve from dosing until last measurable concentration (AUC_last_)	(h) (μM)	542.42 ± 46^†^
Mean residence time (MRT_last_)	(h)	12.1
Area under the curve extrapolated to infinity (AUC_∞_)	(h) (μM)	542.98
Area under the curve due to extrapolation (AUC_%extrap_)	%	0.1

† *Standard error of the mean*.

Additionally, histopathology of spleen, gills, brain, liver, heart, and kidney of the acyclovir-treated koi detected no lesions associated with tissue toxicity when compared to untreated koi in any of the examined timepoints (1, 3, and 6 days). No fish mortalities were observed during the pharmacokinetic analysis.

## Discussion

Recent studies have highlighted the ability of fish to increase humans' psychological and physiological well-being by decreasing stress in general (20) and increasing focus in Alzheimer patients (21). With the reputation of koi carp fish as social and trainable pets that enhance a pond's beauty (22), they have become a vital species needing veterinary care. KHV has been one of the most important threats to koi fish health over the last two decades (23). Therefore, this study aimed to evaluate the potential of acyclovir and cidofovir to be used as antiviral therapy against KHV in koi pet fish.

Results from the cytotoxicity assay suggest both acyclovir and cidofovir are non-toxic to KF1 cells for 24 h at up to 1,500 μM. This contrasts with a previous report that indicated low cytotoxicity of acyclovir above 66.67 μM in the KF1 cell line (10), discrepancy that could be a result of the different cytotoxicity assays used. Nonetheless, comparable to this previous study, the median lethal concentration (LC50) of acyclovir or cidofovir could not be determined. Considering that the acyclovir and cidofovir concentration of 1,500 μM caused a statistically non-significant cytotoxicity of 2.191 ± 2.674% and 2.214 ± 0.8361%, respectively (Figure 2A), concentration which is much higher than the peak plasma concentration of 141 μM obtained in the pharmacokinetic assay for acyclovir (Figure 3), acyclovir poses no concern to koi cells at the expected *in vivo* concentrations.

The cohabitation challenge model indicated that a 10 mg/kg acyclovir intracoelomic dose was capable of decreasing cumulative mortality a mean of 15% when administered after mortalities have been detected during an outbreak (Figure 2A). On the other hand, there was not a significant reduction in cumulative mortality in the cidofovir group (Figure 2A) and that may be explained by the viral DNA polymerase having a potentially narrow specificity for nucleoside analogs. This is something that has been speculated before by Troszok et al. (24) when an 80% decrease in viral load was detected when evaluating the antiviral activity of acyclovir against KHV, but an acyclovir derivative only resulted in a 25% decrease in viral load. Alternatively, it is possible that a 5 mg/kg cidofovir dose is not sufficient for a statistically significant effect on cumulative mortality or that the bioavailability of this drug is poor in koi fingerlings. Regardless, given the efficacy of acyclovir at 10 mg/kg and the current large cost differential between acyclovir and cidofovir (~$0.04 and $4.67/mg, respectively), acyclovir should be prioritized as a good candidate for further study of KHV therapy at this time.

The challenge data also revealed a 3-day delay in initial mortality in the acyclovir-treated group compared to the positive control group (Figure 2A). While this delay was not statistically significant when comparing mean time to first mortality per group (Figure 2B), it is an observation that can be correlated with the obtained pharmacokinetic data (Figure 3). A half-maximal effective concentration (EC50) value has not been reported for acyclovir against KHV in any cell line, therefore the EC50 of acyclovir against Herpes simplex virus 2 in Vero cells (3.99 μM) will be used during this discussion (25). In humans, acyclovir plasma concentrations fall under the EC50 of 3.99 μM in 8 h, which is why twice per day dosing is recommended when administering intravenous infusions (17). With a longer half-life of acyclovir in koi than in humans (14 and 3.16 h, respectively) (17), the acyclovir plasma concentration in koi remained over that threshold of 3.99 μM for 25.6 h. This is also reflected in the very low elimination rate constant of 0.05/h, compared to 0.22/h in humans (26). It is known that EC50 values can vary immensely with viral strain and cell line (11), thus the EC50 for acyclovir against KHV could be lower than 3.99 μM. This would result in the activity of acyclovir being effective against KHV for longer than 25.6 h in koi fingerlings and suggests that a multi-dose protocol may be advantageous and thus warrants further investigation.

The viral load data revealed no significant differences between the acyclovir and positive control groups in both assessed mortalities and survivals (Figure 2C). This is not surprising in mortalities because it is expected to have a viral load comparable to the positive control. On the other hand, it was expected that survivals would have a lower viral load having received acyclovir, as nucleoside analogs halt viral replication. A hypothesis is that a difference in viral load probably occurred early during the challenge, but sampling of survivals was performed at the end of the 30-day period. At this time, the pharmacokinetic data indicates the acyclovir had been cleared from the plasma and the koi immunity response might have naturally decreased the viral load in all group survivals as part of the expected process. This is supported by a statistically significant mean viral load reduction in survivals compared with mortalities (Figure 2C). If future studies were to address the effect of acyclovir in viral load *in vivo*, sampling performed during the time acyclovir is expected to have high plasma levels in koi may be needed.

Lastly, histopathology of targeted organs following a 10 mg/kg acyclovir intracoelomic dose suggest that acyclovir is non-toxic to tissues when used in a short-term protocol at that dose. Tissue histopathology during a longer multi-dose protocol may be needed to assess long-term use toxicity.

In summary, this study provides evidence to support acyclovir as a safe and effective candidate for extralabel treatment of KHV in pet koi fingerlings. It is worth it to mention that age-related differences in drug kinetics may challenge extrapolating this data to adult koi carp (27), so it is a limitation that should be taken into consideration when treating adult koi fish.

## Data Availability Statement

The raw data supporting the conclusions of this article will be made available by the authors, without undue reservation.

## Ethics Statement

The animal study was reviewed and approved by the University of California-Davis' Institutional Animal Care and Use Committee.

## Author Contributions

EQ: visualization. EQ and ES: conceptualization and project administration. EQ, ES, and HK: methodology. EQ, ZY, SY, and DI: formal analysis. EQ, SY, ZY, RH, ES, and HK: investigation. EQ, SY, ZY, ES, DI, and HK: data curation. EQ: writing–original draft preparation. EQ, SY, ZY, RH, ES, DI, and HK: writing–review and editing. ES: supervision. All authors: contributed to the article and approved the submitted version.

## Conflict of Interest

The authors declare that the research was conducted in the absence of any commercial or financial relationships that could be construed as a potential conflict of interest.
